# Primary immunodeficiency diseases in lung disease: warning signs, diagnosis and management

**DOI:** 10.1186/s12931-018-0923-8

**Published:** 2018-11-12

**Authors:** Pere Soler-Palacín, Javier de Gracia, Luis Ignacio González-Granado, Carlos Martín, Carlos Rodríguez-Gallego, Silvia Sánchez-Ramón, Lung ID-Signal Group

**Affiliations:** 1 Hospital U. Vall d’Hebron, P. de la Vall d’Hebron, 119-129, 08035 Barcelona, Spain; 2 Hospital U. 12 Octubre, Av. Cordoba, s/n, 28041 Madrid, Spain; 3 Hospital U. Miguel Servet, P. Isabel la Católica, 1-3, 50009 Zaragoza, Spain; 4 H. Son Espases, C. de Valldemossa, 79, 07120 Palma, Balearic Islands Spain; 5 Hospital Clínico San Carlos, C. del Prof Martín Lagos, s/n, 28040 Madrid, Spain; 6 Hospital Universitari Vall d’Hebron - Institut de Recerca, Barcelona, Spain

**Keywords:** “Immunoglobulins/deficiency”[mesh], “Antibodies/deficiency”[mesh], “Immunoglobulins/administration and dosage”[mesh], “Respiratory Tract Infections”[mesh], “Immunologic Deficiency Syndromes”[mesh]

## Abstract

**Background:**

Pulmonary complications are common in primary immunodeficiency diseases (PID) and contribute to morbidity and mortality in these patients. However, their varied presentation and a general lack of awareness of PID in this setting make early diagnosis and treatment difficult. The aim of this study was to define the warning signs of PID in patients with respiratory manifestations, the necessary diagnostic tests, and the therapeutic management of both children and adults.

**Methods:**

A review of the literature was performed, and 43 PID interdisciplinary specialists were consulted.

**Results:**

This document identifies the pulmonary and extrapulmonary manifestations that should prompt a suspicion of PID, the immunological and respiratory tests that should be included in the diagnostic process according to the level of care, recommendations regarding the use of immunoglobulin replacement therapy according to the specific immunodeficiency, and the minimum recommended immunological and pulmonary monitoring in these patients.

**Conclusions:**

This document is the first to combine scientific evidence with the opinion of a broad panel of experts specializing in the treatment of patients with immunodeficiencies. It aims to provide a useful tool for all practitioners who are regularly involved in the management of these patients.

## Background

Immunodeficiency diseases (ID) are disorders involving a quantitative and/or functional disorder in the immune response [1] that may lead to a greater risk of infections, immune dysregulation, autoimmune phenomena, inflammation, and cancer. These IDs are classified as primary immunodeficiency diseases (PIDs) if their origin is genetic, and secondary (SID) if acquired.

PIDs comprise a heterogeneous group of more than 300 diseases. The Primary Immunodeficiencies Classification Committee of the International Union of Immunological Societies (IUIS) divides PIDs into 8 groups (9, if we include PID phenocopies), depending on their clinical and immunological phenotype [2]. The various congenital or acquired defects can affect both the innate and the acquired immune system [3]. The most common PIDs are predominantly antibody disorders (56.66%), other well defined PIDs (13.91%), phagocytic disorders (8.73%), predominantly T cell deficiencies (7.47%), and complement deficiencies (4.89%) [4].

The prevalence of PID in Europe and the United States ranges from 41 to 83 per 100,000 inhabitants, depending on the study [5, 6]. The diagnosis of PID often emerges from a picture of recurrent or unusual infections, autoimmune diseases, inflammatory processes or cancers. A careful approach must be taken in the early detection of these signs, in order to avoid unnecessary testing, but without delaying diagnosis. However, since their clinical presentation varies widely and understanding of this area is often limited, underdiagnosis and delayed diagnosis are common, generating significant morbidity and mortality [7] and a major social and economic impact [8]. Generally, the early clinical manifestations of PID occur during childhood, but they also are frequently encountered in the second or third decade of life, or even in older patients [9]. Moreover, there are different clinical presentations across various age categories and these presentations in children could widely differ from adult patients with PID [10].

New warning signs alerting to a suspicion of PID are being increasingly described, based on clinical presentations - mainly infectious diseases - and, in some cases, family history. Fifteen years ago, the Jeffrey Modell Foundation (JMF) defined the warning signs of suspected PID in children and adults [8, 11]. This document is a useful basis, but one that needs to be updated, as it does not refer to the autoimmune, inflammatory or malignant manifestations of PIDs, which can be the main clinical presentation in some of these diseases [12]. In addition, a follow-up study by Subbarayan *et at* highlighted the low sensitivity and specificity of these warning signs, recommending that PID awareness initiatives should be mainly targeted at hospital pediatricians and families with a history of PID although these initiatives should also include the general public [13].

Pulmonary complications in PID are common and significantly contribute to the morbidity and mortality of patients. Recurrent respiratory tract infections often constitute the first warning sign in some PIDs and are often the main cause of death in adults with PID [14, 15].

The aim of this study was to define which warning signs should alert to the suspicion of PID in patients with respiratory manifestations, and to develop proposals for intervention in these cases. This document is aimed at primary care (PC) professionals and specialists who see both adult and pediatric patients with respiratory diseases. This study is part of the ID-Signal Project, which aims to generate a series of documents based on the clinical manifestations of IDs dedicated to non-immunologists who may take care of patients with PID or SID in their clinical practice.

## Methods

Initially, an interdisciplinary expert group formed of 2 pulmonologists, 2 immunologists and 2 pediatricians specializing in immunodeficiencies identified items to be addressed in the areas of clinical and laboratory testing, diagnostic imaging, functional tests, and therapeutic approach, and prepared a series of recommendations. A review of the literature was then performed, which acted as a basis for a discussion of the contents of the document in an in-person meeting. The main conclusions and recommendations were forwarded to an external panel of experts for their individual evaluation, depending on their specialty. Although we could have done a Delphi process involving at least two rounds, we preferred a single consultation to have a general view of the opinion of a larger group of experts and to make the process easier. The external panel consisted of 43 experts from all over Spain, with experience in the diagnosis and treatment of PID, working in Spanish National Health System centers. In total, 19 immunologists specializing in adults (adult immunologists, AI), 15 pediatric specialists in immunodeficiencies (pediatric immunologists, PI), 5 pulmonologists taking care of adults (adult pulmonologists, AP), and 4 pediatric specialists in pulmonology (pediatric pulmonologists, PP) participated.

This panel indicated their level of agreement on a scale of 1 to 4, with 1 reflecting “strongly disagree” and 4 “strongly agree”, and they could also add comments when necessary. The results were pooled and the percentages of votes in the categories 1 and 2 (disagree) and in the categories 3 and 4 (agreement) were calculated. Unanimity was determined when 100% of the experts agreed with recommendation/conclusion; consensus, when at least 80% of the experts agreed without unanimity; majority, when more than 65% and less than 80% of the experts agreed with the recommendation/conclusion; and disagreement, when the percentage of agreement was 65% or less.

## Results and discussion

### Lung diseases that should prompt a suspicion of PID

In general, lung complications caused by PID include respiratory tract infections, interstitial lung disease (ILD), and cancers [14]. Recurrent lung infections are often the first warning sign in both adult and pediatric PID patients. These recurrent infections often cause permanent lung damage if appropriate treatment is not promptly initiated. Usually, there are also frequent upper respiratory infections previous to the lower ones, so an optimal pulmonary management should encompass the upper airway [16]. Airway damage can manifest as air trapping, thickening of the bronchial walls, atelectasis and/or bronchiectasis [14, 17]. The pathogens most commonly associated with pneumonia in patients with ID will depend on the type of immunodeficiency presented by the patient but generally include *Streptococcus pneumoniae* and *Haemophilus influenzae*, followed by *Mycoplasma* spp*., Staphylococcus* spp., *Moraxella* spp., *Pseudomonas aeruginosa,* and other viral pathogens, and more rarely *Pneumocystis jirovecii* and various fungi [18].

However, since mortality due to infectious complications has declined, non-infectious complications have begun to account for a large number of the poor clinical outcomes observed in some of these patients [19]. Thus, several histological patterns of ILD have been identified in patients with PID, especially in those with a diagnosis of common variable immunodeficiency disease (CVID), including granulomatous lung disease, lymphoid interstitial pneumonia (LIP), non-specific interstitial pneumonia (NSIP), and organizing pneumonia, and more than one may co-exist in the same patient [14].

The patient profile (adult or pediatric) and pulmonary manifestations (infectious or non-infectious) that lead to a consultation in PC or with a respiratory medicine specialist may be different. Manifestations that should prompt a suspicion of PID are included in Table 1. A respiratory medicine specialist must ensure that pneumonia is confirmed on radiology: this will be suggestive of PID, particularly if disease appears in different sites. Cases of slow-progressing disease must be investigated, and pneumonias that do not resolve with multiple course of antibiotics and require hospital admission, intravenous antibiotics or intensive care assistance, should be noted, along with a high frequency of pneumonias across time (one pneumonia per year for more than one year). Slow-progressing pneumonitis or bronchitis in an infant that requires admission, particularly in the intensive care unit, should be considered as potential manifestations of PID, although severe bronchiolitis may be relatively common in healthy infants.

**Table 1:** Respiratory manifestations indicating a suspicion of PID in primary care and pulmonology clinics

Respiratory Manifestions with suspicion of PID in PC	N (Composition of panel)	Votes in agreement (%)	Degree of agreement
Adult patient			
Recurrent bronchial infections (≥ 2/year), with cough and purulent expectoration	23* (AI, AP)	87.0	**Consensus**
Idiopathic bronchiectasis	23* (AI, AP)	100.0	**Unanimity**
Recurrent pneumonias**	23* (AI, AP)	100.0	**Unanimity**
Chronic bronchial infection	23* (AI, AP)	91.3	**Consensus**
Need for prolonged antibiotic treatment for respiratory infections	23* (AI, AP)	95.7	**Consensus**
Pediatric patient			
Recurrent pneumonias**	19 (PI, PP)	100.0	**Unanimity**
Idiopathic bronchiectasis	19 (PI, PP)	100.0	**Unanimity**
Respiratory manifestations with suspicion of PID in pulmonolgy unit	**N (Composition panel)**	**Votes in agreement (%)**	**Degree of agreement**
Infections			
Adult and pediatric patients			
Recurrent bronchial infections (≥ 2/year), with cough and purulent expectoration	43 (AI, AP, PI, PP)	86.0	**Consensus**
Idiopathic bronchiectasis	43 (AI, AP, PI, PP)	100.0	**Unanimity**
Recurrent pneumonias**	43 (AI, AP, PI, PP)	97.7	**Consensus**
Chronic bronchial infection	43 (AI, AP, PI, PP)	90.7	**Consensus**
Need for prolonged antibiotic treatment for respiratory infections	43 (AI, AP, PI, PP)	93.0	**Consensus**
Pulmonary abscess and pneumatocele	43 (AI, AP, PI, PP)	90.7	**Consensus**
Infections caused by rare or opportunistic microorganisms	43 (AI, AP, PI, PP)	100.0	**Unanimity**
Infants			
Severe infantile bronchiolitis or pneumonia	43 (AI, AP, PI, PP)	93.0	**Consensus**
NON-INFECTIOUS			
Adult and pediatric patients			
Granulomatous-lymphocytic interstitial lung disease	43 (AI, AP, PI, PP)	100.0	**Unanimity**
Bronchiolitis obliterans	43 (AI, AP, PI, PP)	72.1	Majority
Lymphoproliferative syndrome	43 (AI, AP, PI, PP)	90.7	**Consensus**
Alveolar proteinosis	43 (AI, AP, PI, PP)	86.0	**Consensus**
Recurrent serositis	43 (AI, AP, PI, PP)	76.7	Majority
Thymic disorders: thymoma (adult), thymic aplasia (infant)	43 (AI, AP, PI, PP)	100.0	**Unanimity**

AI: adult immunologists; AP: adult pulmonologists; PC: primary care; PI: pediatric immunologists; PID: primary immunodeficiency disease; PP: pediatric pulmonologists

*One value is missing. ** One pneumonia per year for more than one year

### Classification of respiratory diseases by PID type

PIDs have been divided into several categories, according to their clinical characteristics and immune system involvement. In any case, we must remember that inclusion in one category or another involves an important academic component, and in routine practice, the clinical context of the patient will help guide their classification. There are some lung manifestations that should lead to the suspicion of a specific PID. This is the case of pneumatocele and AD-hyperIgE syndrome, bronchiectasis and antibody deficiencies, severe bronchiolitis and severe combined immunodeficiencies or pulmonary fungal infections and phagocyte disorders, among others. However, most lung diseases can occur in a wide range of PID, in which a wide but phased diagnostic protocol is recommended.

Table 2 identifies the respiratory pathogens that are most commonly associated with each type of PID; several manifestations only achieved a majority agreement but not consensus, possibly due to the limited experience available in certain types of manifestations. Discrepancies were found for other manifestations (“abscess and pneumatocele” and “bronchiolitis obliterans”, with agreements of 55.8 and 60.5%, respectively).

**Table 2:** Common respiratory manifestations by PID type

Common respiratory manifestations by PID type	N (Composition of panel)	Votes in agreement (%)	Degree of agreement
Predominantly T cell deficiency (combined and PID-related syndromes)			
Recurrent bronchitis	43 (AI, AP, PI, PP)	72.1	Majority
Idiopathic bronchiectasis	43 (AI, AP, PI, PP)	76.7	Majority
Recurrent pneumonias*	43 (AI, AP, PI, PP)	88.4	**Consensus**
Repeated pneumonia in child	43 (AI, AP, PI, PP)	88.4	**Consensus**
Chronic bronchial infection	43 (AI, AP, PI, PP)	79.1	Majority
Prolonged antibiotic treatment with poor response	43 (AI, AP, PI, PP)	86.0	**Consensus**
Abscess and pneumatocele	43 (AI, AP, PI, PP)	55.8	Discrepancy
Infections caused by rare microorganisms	43 (AI, AP, PI, PP)	100.0	**Unanimity**
Pneumonitis or bronchitis with hospitalization in infants	43 (AI, AP, PI, PP)	86.0	**Consensus**
Interstitial lung disease	43 (AI, AP, PI, PP)	93.0	**Consensus**
Bronchiolitis obliterans	43 (AI, AP, PI, PP)	67.4	Majority
Alveolar proteinosis	43 (AI, AP, PI, PP)	76.7	Majority
Absent thymus or aplasia	43 (AI, AP, PI, PP)	95.3	**Consensus**
Antibody deficiencies			
Recurrent bronchitis	43 (AI, AP, PI, PP)	90.7	**Consensus**
Idiopathic bronchiectasis	43 (AI, AP, PI, PP)	97.7	**Consensus**
Recurrent pneumonias*	43 (AI, AP, PI, PP)	97.7	**Consensus**
Repeated pneumonia in child	43 (AI, AP, PI, PP)	97.7	**Consensus**
Chronic bronchial infection	43 (AI, AP, PI, PP)	90.7	**Consensus**
Prolonged antibiotic treatment with poor response	43 (AI, AP, PI, PP)	97.7	**Consensus**
Pneumonia due to encapsulated bacteria	43 (AI, AP, PI, PP)	97.7	**Consensus**
Interstitial lung disease	43 (AI, AP, PI, PP)	79.1	Majority
Bronchiolitis obliterans	43 (AI, AP, PI, PP)	60.5	Discrepancy
Pulmonary lymphoma	43 (AI, AP, PI, PP)	69.8	Majority
Thymoma	43 (AI, AP, PI, PP)	88.4	**Consensus**
Immune deregulation			
Recurrent bronchitis	43 (AI, AP, PI, PP)	69.8	Majority
Idiopathic bronchiectasis	43 (AI, AP, PI, PP)	74.4	Majority
Chronic bronchial infection	43 (AI, AP, PI, PP)	65.1	Majority
Pneumonia due to encapsulated bacteria	43 (AI, AP, PI, PP)	67.4	Majority
Interstitial lung disease	43 (AI, AP, PI, PP)	81.4	**Consensus**
Phagocyte disorders			
Recurrent pneumonias*	43 (AI, AP, PI, PP)	90.7	**Consensus**
Prolonged antibiotic treatment with poor response	43 (AI, AP, PI, PP)	88.4	**Consensus**
Abscess and pneumatocele	43 (AI, AP, PI, PP)	97.7	**Consensus**
Infections caused by rare microorganisms	43 (AI, AP, PI, PP)	97.7	**Consensus**
Alveolar proteinosis	43 (AI, AP, PI, PP)	69.8	Majority
Innate immunity disorder			
Recurrent pneumonias*	43 (AI, AP, PI, PP)	97.7	**Consensus**
Pneumonia due to encapsulated bacteria	43 (AI, AP, PI, PP)	95.3	**Consensus**
Abscess and pneumatocele	43 (AI, AP, PI, PP)	83.7	**Consensus**
Infections caused by rare microorganisms	43 (AI, AP, PI, PP)	90.7	**Consensus**
Complement deficiency			
Recurrent bronchitis	43 (AI, AP, PI, PP)	65.1	Majority
Idiopathic bronchiectasis	43 (AI, AP, PI, PP)	72.1	Majority
Recurrent pneumonias*	43 (AI, AP, PI, PP)	83.7	**Consensus**
Repeated pneumonia in child	43 (AI, AP, PI, PP)	83.7	**Consensus**
Chronic bronchial infection	43 (AI, AP, PI, PP)	67.4	Majority
Autoinflammatory disease			
Recurrent serositis	43 (AI, AP, PI, PP)	100.0	**Unanimity**

AI: adult immunologists; AP: adult pulmonologists; PI: pediatric immunologists; PID: primary immunodeficiency disease; PP: pediatric pulmonologists

* One pneumonia per year for more than one year

Table 2.

### EXTRAPULMONARY manifestations that should suggest PID

It is very important that the specialist who evaluates a patient with suspected PID due to lung problems also takes in account the presence of extrapulmonary manifestations that might support the clinical suspicion of PID, since one of the main characteristics of PID is its ability to affect multiple organs or systems simultaneously or sequentially. PID may present with skin [20–22], gastrointestinal [23], or autoimmune [24–26] manifestations, among others. Table 3 (adapted from JC Aldave [27]) lists the main extrapulmonary clinical manifestations, with their degrees of agreement. Two common extrapulmonary manifestations did not reach consensus.

**Table 3:** Most common extrapulmonary manifestations of PID (adapted from JC Aldave, JFM) [27]

MOST COMMON EXTRAPULMONARY MANIFESTATIONS OF PID	N (Composition of panel)	Votes in agreement (%)	Degree of agreement
Recurrent or complicated sinusitis	43 (AI, AP, PI, PP)	100.0	**Unanimity**
Recurrent or complicated otitis	43 (AI, AP, PI, PP)	93.0	**Consensus**
Other extrapulmonary infections requiring admission	43 (AI, AP, PI, PP)	88.4	**Consensus**
Ectodermal dysplasia	43 (AI, AP, PI, PP)	72.1	Majority
Chronic diarrhea or malabsorption	43 (AI, AP, PI, PP)	93.0	**Consensus**
Difficult-to-treat giardiasis	43 (AI, AP, PI, PP)	93.0	**Consensus**
Autoimmune cytopenias or other autoimmunity	43 (AI, AP, PI, PP)	93.0	**Consensus**
Lymphadenopathies and hepatosplenomegaly	43 (AI, AP, PI, PP)	97.7	**Consensus**
Related cancers, especially associated with viruses	43 (AI, AP, PI, PP)	83.7	**Consensus**
Family history of ID or consistent manifestations	43 (AI, AP, PI, PP)	88.4	**Consensus**
Post-vaccinal infections	43 (AI, AP, PI, PP)	79.1	Majority

AI: adult immunologists; AP: adult pulmonologists; ID: immunodeficiency; PI: pediatric immunologists; PID: primary immunodeficiency disease; PP: pediatric pulmonologists

On the other hand, there are some histopatological findings which would prompt the clinician to consider a diagnosis of PID before having the suspicious of it. This is the case of granulomas or the absence of plasma cells in a gastrointestinal biopsy.

### Diagnostic tests to be performed in patients with suspected PID

A large number of PIDs can be diagnosed from a detailed clinical history that includes a family history of PID, consanguinity, or family members who died at a young age, a comprehensive physical examination, blood tests, and a determination of serum immunoglobulin (Ig) levels. These tests are available in the vast majority of laboratories, even in PC. The physical examination should be detailed and ordered by body system, and it is important to assess the patient’s nutritional status, with particular attention to height and weight and sequelae from previous infections. The presence of lymphadenopathies or the absence of nodal chains, tonsils, hepatosplenomegaly, etc. must be determined, since in some cases these parameters may orient the professional towards a specific PID. A complete blood count (CBC) with a differential count provides important information on suspected cytopenias (neutropenia, monocytopenia, lymphopenia, or thrombocytopenia) and qualitative cellular changes [9]. The determination of serum Igs (IgG, IgM, IgA, and IgE) is the first step in the evaluation of humoral immunity, and will help diagnose or at least point to a suspicion of quantitative Ig deficiencies, such as congenital agammaglobulinemia, CVID, or selective IgA deficiency, and humoral changes associated with other defects, such as hyper-IgE or hyper-IgM syndrome [9]. On the other side, quantification of calculated globulin (CG, total protein minus albumin [28]), T-cell recombination excision circles (TREC) and kappa-deleting element recombination circles (KREC) may help identify PIDs early in PC. Recently, a study by Pecoraro et al showed that a screening calculated globulin test could be used as a tool to reduce diagnostic delays, improve long-term prognosis and reduce the healthcare costs of antibody deficiency [29].

There are obvious differences between diagnosis in children and adults since, usually, most severe PID are diagnosed in childhood while recurrent respiratory infections, which can be caused by many other non-immunological entities and are the clue for the diagnosis during adulthood. Adolescents are a special population with mixed manifestations. Therefore, first line diagnostic tests in adults are almost specifically based on ruling primary antibody deficiency out (immunoglobulin levels and antibody production assessment), while clinical manifestations will orient the initial tests in childhood (e.g. burst oxidation tests in invasive fungal infections, complement pathway assessment if recurrent bacteremia...).

It is important to assess the results according to the reference values for each age, since there are significant differences which, if not taken into account, can cause PID to be overlooked [30]. In addition, it is always important that the suspected diagnosis is oriented to avoiding unnecessary studies that result in a waste of time and resources. However, when the diagnosis is uncertain and suspicion is high, additional tests, such as functional or molecular studies, are needed, and these must be conducted in reference centers. In fact, some of the warning signs alone (signature signs) as opportunistic infections, or high burden of infection should lead to the referral of the patient to a higher level of care, so that the appropriate tests could be performed.

With regard to tests that are specifically “pulmonary” in a patient with suspected PID, tests adapted to the disease and based on the clinical history are recommended: this is the case for lung function tests and high-resolution computed tomography (HRCT) to evaluate the lung structure, sputum cultures to detect respiratory infections, and sputum cell counts (neutrophils and eosinophils), among others [31]. Chest X-rays may be valuable in acute processes but they are of little use for demonstrating the presence of bronchiectasis or non-infectious pulmonary complications [32, 33]. The sensitivity of lung function tests for the early detection of complications, such as bronchiectasis, is low, and they should always include, if possible, the evaluation of diffusing capacity of the lung for carbon monoxide (DLCO). HRCT, on the other hand, is highly sensitive and specific, and has an important role in the multidisciplinary approach to these conditions, and in the detection, characterization, and quantification of the degree of lung damage, the treatment plan, and follow-up of patients. It is currently the gold standard for the detection and characterization of bronchiectasis [14, 16].

Tables 4 and 5 include immunological and pulmonary tests, respectively, that must be performed when PID is suspected, divided into levels of care. Even though, in the last years the use of next-generation sequencing has become an essential route for diagnosing PID and it is usually performed earlier in the diagnostic algorithm of a patient with suspected PID. Meanwhile, protein and functional testing remain to be tested in reference centers if available. Although all the immunological tests (Table 4) reached consensus, some interesting proposals that the authors agreed to include outside of the table are provided. These include protein expression and genetic studies in the third level for phagocyte deficiencies; determination of IgD levels, C-reactive protein (CRP), erythrocyte sedimentation rate (ESR) in the baseline level for autoinflammatory diseases; serial determination of mevalonate in urine during the acute episode in the second level for autoinflammatory diseases; and replacement of tetanus serologies with tetanus/diphtheria. Pulmonary tests (Table 5) were divided into adults and children. Discrepancies emerged regarding the use of these tests in children, so they could not be validated.

**Table 4:** Immunological tests additional to baseline tests in patients with suspected PID

Baseline level	First level^a^	Second level^b^	Third level^b^	N (Composition of panel)	Votes in agreement (%)	Degree of agreement
Combined						
Complete blood count IgA, IgG, IgM and IgE levels Biochemical analysis^e^	Lymphocyte populations	Extended phenotype, lymphocyte function	Protein expression Functional and genetic studies	34 (AI, AP)	91.2	**Consensus**
PID-associated syndromes						
Complete blood count IgA, IgG, IgM and IgE levels Biochemical analysis^e^	Karyotype ± CGH Array	Studies according to specific suspicion	Protein expression Functional and genetic studies	33* (AI, AP)	87.9	**Consensus**
Antibody production deficiency^c^						
Complete blood count IgA, IgG, IgM and IgE levels Biochemical analysis^e^	Basic antibody production study (ASLO, hemaglutinins and tetanus)	IgG subclasses Response to *Salmonella typhi* or pneumococcus Response to tetanus toxoid^d^ and *H. influenzae* (optional) Lymphocyte populations with extended phenotype B	Protein expression Functional and genetic studies	34 (AI, AP)	91.2	**Consensus**
Immune deregulation						
Complete blood count IgA, IgG, IgM and IgE levels Biochemical analysis^e^	Autoantibody panel (ANA and NOSAB, anti-neutrophils) Targeted hormone study Coombs test Vitamin B12 with neural tube defects Soluble FAS ligand Ferritin Triglycerides Fibrinogen Calcium and phosphorus	Treg Soluble CD25 FoxP3	Protein expression Functional and genetic studies	33* (AI, AP)	97.0	**Consensus**
Phagocyte deficiencies^d^						
Complete blood count IgA, IgG, IgM and IgE levels Biochemical analysis^e^	Oxidation test (DHR) Basic lymphocyte subpopulations	CD18/11b	–	34 (AI, AP)	91.2	**Consensus**
Innate immunity disorder						
Complete blood count IgA, IgG, IgM and IgE levels Biochemical analysis^e^	Studies according to clinical suspicion	–	Protein expression Functional and genetic studies	34 (AI, AP)	97.1	**Consensus**
Complement deficiency						
Complete blood count IgA, IgG, IgM and IgE levels Biochemical analysis^e^	CH50, C3, C4, autoimmunity studies	AP50 Factor quantification	Protein expression Functional and genetic studies	34 (AI, AP)	94.1	**Consensus**
Autoinflammatory diseases^d^						
Complete blood count IgA, IgG, IgM and IgE levels Biochemical analysis^e^	Inflammatory markers CRP ESR	SAA	Genetic studies	33* (AI, AP)	84.8	**Consensus**

AI: adult immunologists; ANA*:* antinuclear antibodies, ASLO: antistreptolysin O; Coombs: antiglobulin test; CRP: C-reactive protein; DHR: dihydrorhodamine 123; DNT: double negative alpha/beta T cells; ESR: erythrocyte sedimentation rate; NOSAB: non-specific autoantibodies; PI: pediatric immunologists; SAA: serum amyloid A; Treg: regulatory T cells

^a^First-level tests will be carried out in primary care or hospital centers which have laboratories with the necessary resources

^b^Second and third-level tests will be carried out in reference centers

^c^ These tests will also be performed in any PID that includes antibody production deficiencies as part of the entity

^d^ Proposals for changes are included throughout the text

^e^ Biochemical analysis includes, at least: blood urea nitrogen, creatinine, liver enzymes, C-reactive protein and albumin

*One value is missing

**Table 5:** Respiratory tests to be conducted in adult (consensus) and pediatric (no consensus) patients with suspected PID

First level^a^	Second level^b^	Third level^c^	N (Composition of panel)	Votes in agreement (%)	Degree of agreement
Adults					
Refer to pulmonologist when warning signs detected	Spirometry* Plethysmography DLCO HRCT +/− abdominal ultrasound Sputum culture (bacteria, mycobacteria, and fungi), if expectoration	Exercise stress tests. Bronchoscopy with bronchoalveolar lavage and transbronchial cryobiopsy. Open lung biopsy or by video-assisted thoracoscopy in case of interstitial lung disease.	5 (AP)	100.0	**Unanimity**
Children					
Spirometry, if possible according to patient age and availability Chest X-ray	Spirometry (technically possible ≥3 years) Plethysmography (≥ 6–7 years, if available) DLCO (≥ 6–7 years) Lung HRCT +/− abdominal ultrasound Sputum culture, if expectoration (induced sputum, if no expectoration)	Lung function studies by age: • In infants: plethysmography; tidal volume determination; tidal or raised volume rapid thoracoabdominal compression technique. • In preschoolers: oscillometry; oscillatory resistance. Bronchoscopy with bronchoalveolar lavage, in the case of localized or diffuse bronchiectasis or interstitial lung disease, even if patient is expectorating. Open lung biopsy or video-assisted thoracoscopy in case of interstitial lung disease Transbronchial cryobiopsy in children > 7 years as alternative to lung biopsy	4 (PP)	50.0	Discrepancy

AP: adult pulmonologists; DLCO: diffusing capacity of the lung for carbon monoxide; HRCT: high-resolution computed tomography; PP: pediatric pulmonologists

^a^First-level tests will be carried out in PC centers

^b^ First-level tests will be carried out by respiratory specialists

^C^ Third-level tests will be carried out immunologists or in reference center

* Spirometry may be performed as part of level 1 when available at the primary care setting

### Immunoglobulin replacement therapy in PID

Immunoglobulin replacement therapy (IGRT) is essential in patients with PIDs that directly affect B cell function and antibody production, primarily in severe cases, but also in patients in whom involvement is not obvious from routine testing [34]. IGRT has brought about a significant change in the prognosis of patients with PID. The mortality of CVID has fallen from 29% in 1971 (before the introduction of intravenous Ig) to 19.6% in 2012 [35]. In pulmonary complications of PID, IGRT has halved the number of bacterial pneumonias [36]; however, this treatment does not appear to improve other recurrent respiratory infections [36].

There are currently 6 PID phenotypes for which IGRT is indicated or may be indicated [37]: agammaglobulinemia due to the absence of B cells; hypogammaglobulinemia with impaired specific antibody production; selective antibody deficiencies (IGRT use must be individually evaluated); hypogammaglobulinemia with normal-quality antibody response; isolated deficiency of an IgG subclass with recurrent infections; and recurrent infections due to an unknown immune mechanism.

Since IGRT was introduced in the 1980s, the administered dose of Ig has been gradually increased to achieve higher IgG plasma concentrations [38]. For example, in the case of patients with CVID, IgG plasma concentrations to prevent bacterial infections may range between 5 and 17 g/L, although trough concentrations of IgG of around 8–10 g/L are often recommended. These levels can be achieved with a dose range of 0.2–1.2 g/kg/month. Doses must be individualized, and higher doses are required in patients with bronchiectasis and those with certain phenotypes [38].

IGRT can be administered by 2 routes: subcutaneous (SCIG) and intravenous (IVIG); the muscular route has fallen into disuse. There is also a facilitated SCIG which improves its bioavailability and allows infusions every 3–4 weeks [39]. Adequate doses administered by both routes provide physiological IgG concentrations [40, 41], although the kinetics are different: intravenous administration produces a rapid increase in IgG plasma concentrations, while subcutaneous administration produces more gradual increases in plasma levels [41, 42] and more stable serum concentrations [40, 43]. SCIG can be particularly beneficial in patients with low IgG concentrations despite intravenous treatment, since higher mean serum values can be achieved with lower doses of immunoglobulin [44–46]. Both routes of administration have shown equivalence in terms of efficacy and safety [37, 45, 47], although the incidence of serious systemic adverse effects is higher with IVIG and more mild local effects are observed with SCIG [42, 45, 48].

Different factors must be considered when selecting the route of administration, as not all patients benefit equally from both [49]. Firstly, SCIG is associated with a better quality of life for both patients and their caregivers [46, 50, 51]. A review of 25 studies showed improved health-related quality of life in patients who switched IVIG administration in the hospital for home-administered SCIG [45]. This same review also concluded that SCIG therapy was more cost-effective, mainly due to the reduction in days missed from school or work [45]. The subcutaneous route is also more appropriate for patients with difficulties for venous access, and does not appear to adversely affect renal function [37, 41]. However, the patient must be trained, and the equipment and infrastructure required for correct administration may not be easily achieved for all patients. Finally, patient preferences are fundamental when decisions are being made. Multidisciplinary long-term monitoring programs must be implemented that are flexible regarding the alternate use of both routes, depending on the life circumstances of each patient [52, 53]. Despite all, the decision of which route and location of therapy is best may change with age and personal circumstances and requires ongoing re-evaluation; however patients who are stable and managing well with a specific route should not be swapped onto different products for non-clinical reasons.

### Monitoring PID patients with pulmonary involvement

Survival and quality of life of patients with certain PIDs has improved significantly in recent years, particularly as a result of better diagnosis and treatment of infections and other comorbidities, the development of highly potent antimicrobial agents, and the development of specific therapeutic alternatives, such as IgG and others [54]. However, we must not allow the reduction in the number of infections and hospitalizations to generate a lower perception of severity that might lead to a relaxation in the regular clinical monitoring of the disease [9].

The decision on the frequency of evaluations of PID patients depends on several factors, including the type of PID, clinical conditions, and age. There is currently no internationally agreed consensus on the follow-up of patients with PID and lung involvement, but it has been suggested that patients should be evaluated at least once every 6–12 months with a full clinical history and physical examination [9]. Whenever possible, this evaluation should be performed by an expert in PID [55].

The literature reviewed proposes that the follow-up of patients with pulmonary complications should include serial lung function tests and imaging studies, along with regular sputum cultures [9, 56]. Chest X-ray will only be indicated if acute pulmonary involvement is suspected. It is recommended that HRCTs are performed every 3–5 years in patients under treatment, depending on their clinical stability [57].

Table 6 sets out the recommendations on the minimum tests to be performed during the clinical monitoring of the PID patient with pulmonary symptoms.

**Table 6:** Minimum tests to be performed during monitoring of the patient with PID and respiratory symptoms

TESTS	N (Composition of panel)	Votes in agreement (%)	Degree of agreement
Patient visit every 6–12 months.	34 (AI, AP)	91.2	**Consensus**
Immunological studies (complete blood count, biochemistry with LDH and Igs) every 6–12 months.	34 (AI, AP)	91.2	**Consensus**
In the event of RT with gammaglobulins, IgG trough values must be measured more frequently, at least during dose adjustment.	34 (AI, AP)	100.0	**Unanimity**
Respiratory tests: • Annual lung function tests are recommended every year. • Spirometry must be performed every 4–6 months in the absence of lung disease. • The sputum culture must be performed at each visit if patient is expectorating and in case of any exacerbation.	9 (AP, PP)	100.0	**Unanimity**
Lung CT every 2–3 years if the patient has pulmonary involvement and every 5 years otherwise. Radiation must be minimized in case of radiosensitive ID.	9 (AP, PP)	100.0	**Unanimity**
Extrapulmonary tests should be performed according to the patient’s symptoms or manifestations.	43 (AI, PI, AP, PP)	97.7	**Consensus**

AI: adult immunologists; AP: adult pulmonologists; ID: immunodeficiency; Ig: immunoglobulins; LDH: lactate dehydrogenase; PI: pediatric immunologists; PID: primary immunodeficiency disease; PP: pediatric pulmonologists; RT: replacement therapy

## Conclusions

This study is the first to focus on PID from a pulmonary point of view, addressing the diagnosis, treatment and follow-up of both adult and pediatric patients, while simultaneously including the opinion and clinical experience of more than 40 experts from different fields and specialties who regularly treat these deficiencies. This document is intended to be a useful tool for both primary care professionals and specialists who are treating patients with lung disease, helping them to identify the warning signs of PID in order to make an early diagnosis or else to refer the patient to a specialized unit, thus avoiding unnecessary complications. Other approaches that may help an early identification of PIDs should include education and awareness for specialists. Finally, future possible shortcomings of the study will involve other medical specialties and other clinical manifestations of IDs.

## Abbreviations

AI: Adult immunologists
ANA: Antinuclear antibodies
AP: Adult pulmonologists
ASLO: Antistreptolysin O
CG: Calculated globulin
COOMBS: Antiglobulin test
CRP: C-reactive protein
CVID: Common variable immunodeficiency
DHR: Dihydrorhodamine 123
DLCO: Diffusing capacity of the lung
DNT: Double negative alpha/beta T cells
ESR: Erythrocyte sedimentation rate
HRCT: High-resolution computed tomography
ID: Immunodeficiency
Ig: Immunoglobulins
IGRT: Immunoglobulin replacement therapy
ILD: Interstitial lung disease
IUIS: International Union of Immunological Societies
IVIG: Intravenous Ig
JMF: Jeffrey Modell Foundation
KREC: Kappa-deleting element recombination circles
LDH: Lactate dehydrogenase
NOSAB: Non-organ-specific autoantibodies
NSIP: Non-specific interstitial pneumonia
PC: Primary care
PI: Pediatric immunologists
PID: Primary immunodeficiency disease
PP: Pediatric pulmonologists
RT: Replacement therapy
SAA: Serum amyloid A
SCIG: Subcutaneous Ig
SID: Secondary immunodeficiency diseases
TREC: T-cell recombination excision circles
Treg: Regulatory T cells
